# A guide to building the matrisome interactome: from computational predictions to experimental validation

**DOI:** 10.1111/febs.70344

**Published:** 2025-11-19

**Authors:** Leanna Leverton, Amanpreet Kaur Bains, Ikram Isa, Sylvie Ricard‐Blum, Alexandra Naba

**Affiliations:** ^1^ Department of Physiology and Biophysics University of Illinois Chicago IL USA; ^2^ ICBMS, UMR 5246 CNRS – Université Claude Bernard Lyon 1 Villeurbanne France; ^3^ University of Illinois Cancer Center Chicago IL USA

**Keywords:** extracellular matrix, interaction network, protein complexes, protein domains, protein–protein interactions

## Abstract

The extracellular matrix (ECM) is a complex meshwork of proteins and polysaccharides found in all multicellular organisms, that provides structural support to cells and organize them into tissues and organs. In addition to its architectural role, the ECM conveys key mechanical and biochemical signals to cells via cell surface receptors that activate biological pathways controlling a plethora of cellular behaviors, including proliferation, stemness, adhesion, migration, or differentiation. The structural integrity of the ECM and its signaling functions are mediated by protein–protein and protein–polysaccharide interactions. Uncovering the mechanisms regulating these interactions is critical to better understand ECM assembly and biology and envision therapeutic strategies targeting the ECM. Here, we provide a comprehensive review of the computational and experimental approaches available to identify and characterize ECM protein interactions, from individual interactions to the interactome of the full matrisome. We first review computational tools currently available to predict interactions and then describe techniques that allow the experimental determination of these interactions and the parameters governing them. Using examples from original research literature, we illustrate how each approach has been applied to identify ECM interactions and has helped advance our understanding of ECM functions in health and disease. To assist researchers interested in the field, we propose a roadmap combining computational and experimental approaches to generate cell‐, tissue‐, or disease‐specific ECM interaction networks. Lastly, we discuss the remaining challenges and perspectives in the field of ECM interactomics.

## Graphical abstract

This guide proposes a roadmap combining state‐of‐the‐art computational and experimental approaches (including biophysical, biochemical, and imaging techniques) to identify, characterize, and contextualize extracellular matrix (ECM) protein interactions. Defining the interactome of ECM proteins in the context of health and disease can provide insights into ECM biology and enable the discovery of previously undescribed therapeutic targets.

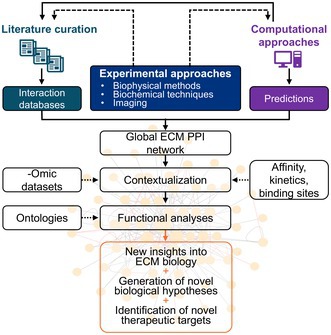

**Abbreviations**
AP‐MS: affinity purification coupled to mass spectrometry
BLI: biolayer interferometry
BMP‐1: bone morphogenetic protein 1
ECM: extracellular matrix
FN: fibronectin
FRET: Förster resonance energy transfer
GAG: glycosaminoglycan
GST: glutathion S‐transferase
HA: hemagglutinin
IP: immunoprecipitation
ITC: isothermal titration calorimetry
LC–MS/MS: liquid chromatography coupled to tandem mass spectrometry
MMP: matrix metalloproteinase
MS: mass spectrometry
MST: microscale thermophoresis
PL‐MS: proximity labeling coupled to mass spectrometry
PPI: protein–protein interaction
QCM‐D: quartz crystal microbalance with dissipation monitoring
scRNA‐Seq: single‐cell RNA sequencing
SPR: surface plasmon resonance
SPRi: surface plasmon resonance imaging
TIMP: tissue inhibitor of metalloproteinase
Y2H: yeast two‐hybrid

## Introduction

**Fig. 1:**
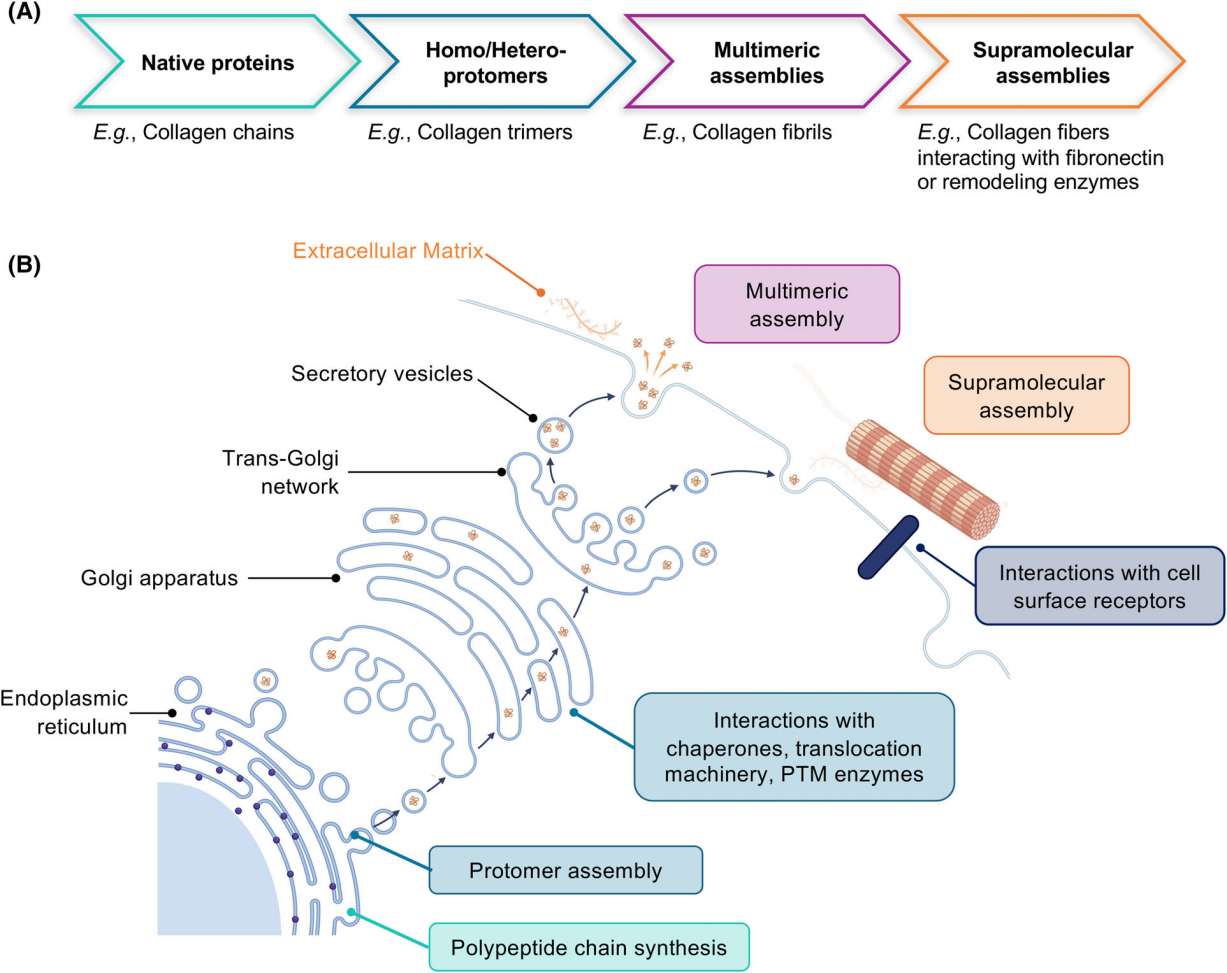
ECM protein interactions span scale, space, and time. (A) ECM assembly results from a multi‐scale process. ECM proteins are synthesized as polypeptide chains. Some of them (*e.g*., collagens, laminins, thrombospondins, and tenascins) form homo‐ or hetero‐protomers (*i.e*., multimers) that constitute the functional building block of higher‐order supramolecular assemblies (*e.g*., fibrils for collagens) often in association with other ECM components including proteins, proteoglycans, or GAGs. Here, collagens are used to illustrate the process of ECM assembly. (B) Schematic representation of the localization of protein–protein interactions of ECM proteins through the intracellular biosynthetic and secretory pathways, at the cell membrane, and in the extracellular space.

The extracellular matrix (ECM) is a complex meshwork of proteins and polysaccharides that constitutes the architectural scaffold of all tissues [1, 2, 3]. Using sequence homology, we have previously defined that the matrisome is composed of over 1000 genes in mammals and comprises both core components, including collagens, proteoglycans, and other ECM glycoproteins, and associated components that can modulate the structural, physical, mechanical, and signaling properties of the ECM [2, 4]. The assembly and structural integrity of the ECM are governed by tightly regulated biomolecular interactions that span a broad molecular scale (Fig. 1A). Structural ECM proteins are synthesized as single chains, and most assemble into homo‐ or hetero‐protomers, which are the initial building blocks of complex ECM structures, such as collagen trimers [5], laminin trimers [6], fibronectin (FN) dimers [7], or tenascin C hexamers [8]. These protomers then assemble into multimers and eventually into supramolecular assemblies involving other proteins or proteoglycans (Fig. 1A). Interactions involving ECM proteins are dynamic and span time and space (Fig. 1B). ECM protein–protein interactions (PPIs) occur intracellularly during ECM protein biosynthesis, when ECM proteins undergo posttranslational modifications, protomeric assembly, and trafficking through the secretory pathway [1]. ECM proteins interact during this process with a diverse array of posttranslational modifying enzymes (*e.g*., glycosylases, hydroxylases) and chaperones to ensure their proper folding, secretion, and functions [1, 9, 10, 11, 12, 13, 14]. Extracellularly, ECM PPIs ensure the structural assembly of the ECM scaffold [15, 16, 17, 18]. For some proteins, this process necessitates interactions between ECM proteins and ECM receptors at the cell surface, including integrins, as extensively documented for fibronectin, one of the most abundant and ubiquitous ECM glycoproteins [19, 20].

The ECM scaffold is dynamic and undergoes physiological remodeling to sustain tissue needs and maintain homeostasis. This process is under the tight control of ECM‐remodeling enzymes, such as the matrix metalloproteinases (MMPs), a disintegrin and metalloproteinases (ADAMs), a disintegrin and metalloproteinase with thrombospondin motifs (ADAMTSs), or cathepsins, which are enzymes that cleave ECM proteins and generate matricryptins or matrikines that are cryptic fragments capable of signaling functions [21]. On the other hand, enzymes of the lysyl oxidase (LOX) or transglutaminase families [22, 23, 24] can remodel the ECM scaffold by crosslinking ECM proteins. Lastly, the signaling functions of ECM proteins rely on finely tuned interactions engaging ECM proteins and their receptors, as well as ECM proteins and growth factors [25]. ECM‐mediated signaling functions control a broad range of cellular functions, across the lifespan, from embryonic development to aging [3, 26, 27], including proliferation [28], stemness, differentiation [29], adhesion, and migration [30].

At the molecular level, PPIs are mediated by the recognition and binding of protein domains that are portions of amino acid sequences that adopt a defined conformation [31], short amino acid motifs, such as the RGD and LDV motifs found in numerous ECM proteins and which constitute the binding sites for several integrins [32], or conformational binding sites.

Because of their critical roles, any disruption of ECM PPIs has significant consequences on the properties of the ECM and can lead to diseases. For example, the regions of the helical domain of collagen I, shown to be major binding sites for integrins, metalloproteinases, fibronectin, and proteoglycans, are hotspots for lethal mutations reported in osteogenesis imperfecta patients, highlighting the critical roles of these interactions [33, 34]. Excessive ECM deposition is a hallmark of cancer [35] and fibrosis [36], and it is likely that these diseases trigger a rewiring of ECM protein interactions due to changes in abundance [37], PTMs, or conformation resulting from sequence variants.

This review presents the computational resources and biophysical, biochemical, and *in situ* approaches that can be used to identify and characterize ECM protein–protein interactions. We discuss the limitations of existing methods and technical challenges that have hindered, so far, the high‐throughput profiling of the interactome of insoluble ECM proteins and propose possible avenues to move the field of ECM interactomics forward. Of note, all the methods described in this review can be used to probe interactions involving soluble proteins, including soluble proteins found in the extracellular space, such as growth factors, matrikines released upon ECM remodeling, and moonlighting proteins, that is, proteins with localization‐dependent function including intracellular proteins. While we will primarily focus on methods to characterize ECM PPIs, we must mention that interactions between proteins and glycosaminoglycans (GAGs) are also critical to the structure and function of the ECM *in vivo*, and will be discussed as future directions.

## Representation of ECM protein interactions in public databases

Many resources compile information on protein sequences and structures, which are key to understanding PPIs. These resources include the Universal Protein Knowledgebase (UniProt, [38]), InterPro [39], the Protein Data Bank (PDB, [40]), the Class, Architecture, Topology, and Homologous superfamily (CATH) protein structure classification database [41], or the Structural Classification of Proteins extended (SCOP) database [42] (Table 1). In addition, some resources provide specific information on ECM proteins, such as the Matrisome portal [43], MatrisomeDB [44], basement membraneBASE [45], the laminin database [46], phylobone [47], or the now archived mouse basement membrane bodymap [48] (Table 2).

**Table 1:** Resources and databases on protein sequences and structures.

Resource	Description	Link	References
Universal Protein Knowledgebase (UniProt)	Provides information on protein name, taxonomy, amino acid sequence, domains, structure, variants, phenotypes, PTMs, processing, subcellular location, functions, and expression. Extensive cross‐referencing to other databases	https://www.uniprot.org/	Bateman *et al*., 2025 [38]
InterPro	Provides information on protein families, domains, motifs, and functional sites	https://www.ebi.ac.uk/interpro/	Blum *et al*., 2025 [39]
The Protein Data Bank (RCSB PDB)	Provides experimentally determined 3D structures from the PDB archive, integrative 3D structures from the PDB, and computed structure models (CSM) from AlphaFold B and ModelArchive	https://www.rcsb.org/	Burley *et al*., 2025 [40]
Classification, Architecture, Topology, and Homologous superfamily (CATH) protein structure classification database	Provides hierarchical information on protein structures downloaded from the Protein Data Bank (PDB) based on Class (C), Architecture (A), Topology/fold (T), and Homologous superfamily (H).	https://www.cathdb.info/	Sillitoe *et al*., 2021 [41]
Structural Classification of Proteins (SCOP) database	Provides information on structural and evolutionary relationships between all proteins of known structure	https://www.ebi.ac.uk/pdbe/scop	Andreeva *et al*., 2020 [42]

**Table 2:** Resources and databases on ECM protein sequences, structure, and localization.

Resource	Description	Link	References
The Matrisome Portal	Provides lists of matrisomes of multiple model organisms, defined *in silico* through protein sequence analysis	https://matrisome.org/	Naba *et al*., 2016 [43]
MatrisomeDB	Provides a searchable database of curated proteomic datasets on the ECM of human and mouse samples	https://matrisomedb.org	Shao *et al*., 2023 [44]
Basement membraneBASE	Provides an atlas of basement membrane composition in development, adult life, disease, and across animal species, formed by connecting basement membrane gene networks with protein localization data. Protocols for studying basement membranes	https://bmbase.manchester.ac.uk/	Jayadev *et al*., 2022 [45]
Laminin database	Provides information on the laminin family in health and disease, including neuromuscular disorders and the miRNA‐laminin relationship	http://www.lm.lncc.br/	Golbert *et al*., 2014 [46]
Phylobone	Provides a database of bone ECM proteins in humans and model organisms	https://phylobone.com/	Fontcuberta‐Rigo *et al*., 2023 [47]
The Mouse Basement Membrane Bodymap (archived)	Provides body‐wide localizations of basement membrane proteins in developing mouse embryos	https://dbarchive.biosciencedbc.jp/archive/matrixome/bm/home.html	Manabe *et al*., 2008 [48]

In addition to these databases and portals, other databases described in Tables 3 and 4 report protein–protein interactions. These include the International Molecular Exchange (IMEX) consortium database [49], and databases of members of this consortium, including IntAct [50], the Complex Portal [51], the Molecular INTeraction database [52], the Integrated Interaction Database (IID, [53]), BioGRID [54], STRING [55], the Human Reference Interactome (HuRI, [56]), the database of three‐dimensional interacting domains (3did, [57]), Protein–Protein Interactions Domain Miner (PPD, [58]), the BioPlex interactome [59], the Structural Database of Kinetics and Energetics of Mutant Protein Interactions (SKEMPI, [60]), and the Protein Common Interfaces Database (ProtCID, [61]). ECM‐specific interactions databases include MatrixDB [62], GAG‐DB [63], and the adhesome portal [64] and are described in Table 4.

**Table 3:** Protein–protein interaction databases.

Resource	Description	Link	References
IMEx Consortium	Manually curated database of non‐redundant physical molecular interaction data from a broad taxonomic range of organisms	https://www.imexconsortium.org/	Porras *et al*., 2020 [49]
IntAct (IMEx Consortium member)	Manually curated database of molecular interactions, derived from the scientific literature and direct data depositions. Captures the experimental detail essential for the interpretation of molecular interaction data. Curated by partners of the IMEx consortium, for which the IntAct database provides a shared curation and dissemination platform. Uses IMEx curation rules. Has a built‐in network visualization tool	https://www.ebi.ac.uk/intact/home	Del Toro *et al*., 2022 [50]
Complex Portal	Resource for macromolecular complexes from several key model organisms. In addition to the expert‐manually curated complexes, the portal now holds high‐confidence machine‐learning‐predicted human complexes from hu.MAP3.0 and MuSIC. It includes multimeric native ECM proteins (e.g., collagens, laminins, thrombospondins) and their dimeric receptors (integrins)	https://www.ebi.ac.uk/complexportal/home	Balu *et al*., 2025 [51]
The Molecular INTeractions (MINT) Database (IMEx Consortium member)	Manually curated protein interaction data from the scientific literature. Uses IMEx curation rules	https://mint.bio.uniroma2.it/	Calderone *et al*., 2020 [52]
Integrated Interactions Database (IID) (IMEx Consortium member)	A database of experimentally detected PPIs from 10 curated databases, orthologous PPIs, and high‐confidence PPIs predicted by state‐of‐the‐art computational methods. Provides context‐specific PPI networks (*e.g*., tissues, subcellular localizations, diseases, and druggability status)	https://iid.ophid.utoronto.ca/	Kotlyar *et al*., 2022 [53]
BioGRID	Database of protein, genetic, and chemical interactions, including many known drugs. Has a built‐in network visualization tool	https://thebiogrid.org/	Oughtred *et al*., 2021 [54]
STRING	Database of physical interactions and functional associations from various sources: experimental interactions aggregated from primary databases, computationally predicted protein–protein interactions (gene neighborhood, fusions, and co‐occurrence), and others (text mining, co‐expression, and protein homology)	https://string‐db.org/	Szklarczyk *et al*., 2023 [55]
The Human Reference Interactome (HuRI)	Resource for protein–protein interactions (PPI) identified using yeast two‐hybrid screens and PPIs of comparable high‐quality data extracted from the scientific literature	http://www.interactome‐atlas.org/	Luck *et al*., 2020 [56]
The Protein Data Bank via the Research Collaboratory for Structural Bioinformatics (RCSB PDB)	Database for three‐dimensional (3D) structures of protein complexes	https://www.rcsb.org/	Burley *et al*., 2025 [40]
The database of three‐dimensional interacting domains (3did)	Catalog of PPIs for which a high‐resolution 3D structure is known. 3did collects and classifies all structural templates of domain–domain interactions in the Protein Data Bank, providing molecular details for these interactions	https://3did.irbbarcelona.org/	Mosca *et al*., 2014 [57]
Protein–Protein Interactions Domain Miner (PPDM)	Manually curated database of domain‐domain physical interactions, predicted interactions, and complex structures	http://ppidm.loria.fr	Alborzi *et al*., 2021 [58]
The BioPlex Interactome	Database with two proteome scale, cell‐line‐specific PPI networks in HEK 293 T and HCT116 cells. Interactions are identified by affinity purification–mass spectrometry. The BioPlex interactome is now available in the IntAct database	https://bioplex.hms.harvard.edu/	Geistlinger *et al*., 2023 [59]
Structural database of Kinetics and Energetics of Mutant Protein Interactions (SKEMPI)	Database of binding free energy changes upon mutation for structurally resolved protein–protein interactions (e.g., MMP1/TIMP‐1 inhibitor)	https://life.bsc.es/pid/skempi2	Jankauskaite *et al*., 2019 [60]
Protein Common Interfaces Database (ProtCID)	A database for structural information on protein and protein domain interactions	https://dunbrack2.fccc.edu/ProtCID/	Xu *et al*., 2020 [61]

**Table 4:** ECM and adhesion interaction databases.

Resource	Description	Link	References
The ECM interaction database MatrixDB (IMEx Consortium member)	Manually curated database of experimentally validated interactions of ECM proteins and glycosaminoglycans. Also includes predicted ECM interactions from the Integrated Interactions Database (IID). Uses IMEx curation rules	https://matrixdb.univ‐lyon1.fr/	Samarasinghe *et al*., 2025 [62]
GAG‐DB	Manually curated database for structures of GAG‐protein complexes determined by X‐ray, NMR, and scattering data, often associated with molecular modeling	https://gagdb.glycopedia.eu/	Pérez *et al*., 2020 [63]
The Adhesome: A Focal Adhesion Network	Manually curated protein–protein interaction network developed from the scientific literature. The network comprises known interactions and cellular components constituting the focal adhesion complex in mammalian cells	https://adhesome.org/	Winograd‐Katz *et al*., 2014 [64]

These resources can be queried to gain information on the nature, parameters, and functional relevance of PPIs. We encourage the readers to consider the type of data provided by those resources (*e.g*., experimental data curated by experts, and/or predictions using various approaches) to handle the data retrieved from those databases correctly.

**Fig. 2:**
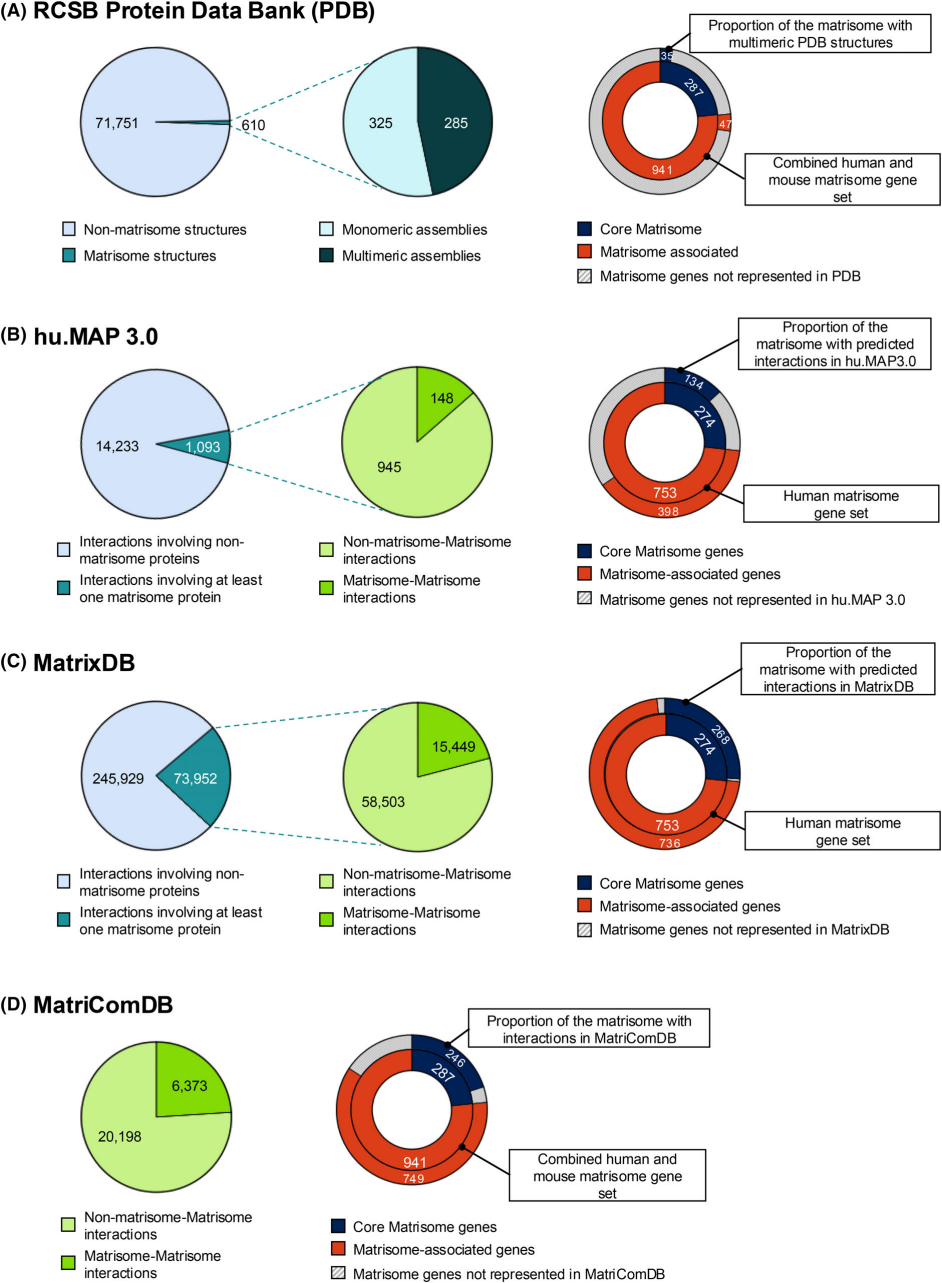
Representation of Matrisome protein interactions in various databases. (A) *Left panel*: Pie chart represents the proportion of structures corresponding to non‐matrisome (light blue) and matrisome entries (teal) in RCSB PDB. *Middle panel*: Pie chart illustrates the distribution of matrisome entries as monomeric structures (light teal) and structures of multimeric protein assemblies involving at least one matrisome protein (dark teal). *Right panel*: The inner donut chart represents the composition of the combined human and mouse matrisome gene set, which is divided into core matrisome (indigo) and matrisome‐associated (orange) genes. The outer donut chart depicts the proportion of core matrisome and matrisome‐associated genes for which at least one multimeric assembly structure is cataloged by RCSB PDB. (B) *Left panel*: Pie chart represents the proportion of interactions involving non‐matrisome proteins (light blue) and at least one matrisome protein (teal) predicted in hu.MAP3.0. *Middle panel*: Interactions are further classified into those involving one matrisome protein and one non‐matrisome protein (green) and those involving two matrisome proteins (lime green). *Right panel*: The inner donut chart represents the composition of the human matrisome gene set, which is divided into core matrisome (indigo) and matrisome‐associated (orange) genes. Outer donut chart depicts the proportion of core matrisome and matrisome‐associated genes for which there is at least one predicted interaction in hu.MAP3.0. (C) *Left panel*: Pie chart represents the proportion of interactions involving non‐matrisome proteins (light blue) and at least one matrisome protein (teal) listed in MatrixDB (version 4.0, 2024). *Middle panel*: Interactions are further classified into those involving one matrisome protein and one non‐matrisome protein (green) and those involving two matrisome proteins (lime green). *Right panel*: The inner donut chart represents the composition of the human matrisome gene set, which is divided into core matrisome (indigo) and matrisome‐associated (orange) genes. The outer donut chart depicts the proportion of core matrisome and matrisome‐associated genes for which at least one interaction is listed in MatrixDB. Note here that the interactions of complexes and matrikines were mapped to the corresponding genes. (D) *Left panel*: Interactions compiled in MatriComDB are classified into those involving one matrisome protein and one non‐matrisome protein (green) and those involving two matrisome proteins (lime green). *Right panel*: The inner donut chart represents the composition of the human matrisome gene set, which is divided into core matrisome (indigo) and matrisome‐associated (orange) genes. The outer donut chart depicts the proportion of core matrisome and matrisome‐associated genes for which at least one interaction is listed in MatriComDB.

Here, we sought to determine how well‐represented ECM PPIs are in two global databases compiling information on protein complexes: the Protein Data Bank (PDB) [40], which provides experimental structures of protein complexes, and huMAP3.0 [65], a database of protein complexes predicted using machine learning by a model trained on over 25 000 mass spectrometry (MS) datasets obtained using different approaches including co‐fractionation‐MS (CF‐MS), affinity purification coupled to ‐MS (AP‐MS), and proximity labeling (*see below*). Interrogation of the content of the Protein Data Bank via the Research Collaboratory for Structural Bioinformatics (RCSB) interface [40] contains a very limited number of experimental structures of ECM components, and most of them are of individual protein domains or pairs of domains, but not of full‐length proteins. Specifically, of the 72 361 human and mouse entries listed in PDB, only 610 pertain to matrisome components (Fig. 2A, left panel). Of these, only 285 are structures of multimeric protein assemblies involving at least one matrisome protein (Fig. 2A, middle panel). In aggregate, these structures provide information on 35 core matrisome proteins and 47 matrisome‐associated proteins (Fig. 2A, right panel). In aggregate, we found that PDB reports structures on the products (fragments, full‐length protein, complexes) of 91 genes, or 8.9%, of the human matrisome.

Our interrogation of hu.MAP.3.0 found that 1093 out of the 15 326 predicted complexes listed in this database involve at least one matrisome component (Fig. 2B, left panel). In terms of coverage, these interactions involve 134 of the 274 canonical core matrisome proteins and 398 of the 753 canonical matrisome‐associated proteins, so about 50% of the matrisome (Fig. 2B, right panel). However, these predictions must now be confirmed experimentally.

In addition to these global resources, we have developed MatrixDB, a manually curated database of experimentally validated ECM interactions, including ECM PPIs, interactions between ECM proteins and GAGs, or ions. MatrixDB also includes interactions predicted with high confidence [62]. Of the 319 881 predicted and experimentally validated interactions related to human proteins listed in MatrixDB, 73 952, or 23%, involve at least one matrisome protein (Fig. 2C, left panel), and about 20% of these are interactions between two matrisome components (Fig. 2C, middle panel). Furthermore, MatrixDB reports at least one interaction for nearly 98% of the human matrisome (Fig. 2C, right panel).

## Computational tools available to predict protein interactions

### Inferring protein interactions from gene co‐expression

The advent of single‐cell RNA sequencing (scRNA‐Seq) has prompted computational biologists to develop algorithms to infer protein interactions, specifically ligand/receptor pairs and resulting downstream signaling, from co‐expression data. Representation of the matrisome in the databases powering these tools is minimal. For example, CellTalkDB compiles 3398 interactions, but only 98 involve two matrisome components, and only six involve two core matrisome components [66]. CellChatDB v2 contains 3234 interactions, of which only 129 involve two matrisome components and none report core matrisome‐core matrisome PPIs [67], whereas NicheNet v2 reports 12 659 interactions with 113 involving two matrisome components, and only two involving two core matrisome components [68]. In light of these observations, we thought to devise a tool specifically designed to infer ECM‐ECM and cell‐ECM communication systems from scRNA‐Seq datasets [69]. MatriCom is powered by MatriComDB, a database built by assembling both experimental and predicted interactions involving at least one matrisome protein from six databases, including MatrixDB [62], basement membraneBASE [45], a subset of the Kyoto Encyclopedia of Genes and Genomes (KEGG), interactions listed under the physical subnetwork of STRING [55], BioGRID [54], and OmniPath [70]. We further assigned a confidence score to each interaction based on its robustness and imposed some constraints; for example, single collagen or laminin chains need to be expressed by the same cell to form a functional trimer. *In toto*, MatriComDB contains over 26 000 unique interactions, of which over 6000 are between two matrisome proteins (Fig. 2D, left panel), and provides at least one interaction for 995 (or > 80%) of the human and mouse matrisomes combined (Fig. 2D, right panel).

However, this type of approach presents critical limitations. First, there are now well‐documented discrepancies between gene expression levels and protein abundance [71, 72]. In addition, gene‐level approaches provide binary interaction data between gene products (*i.e*., polypeptide chains) and hence cannot capture interactions established by multimeric ECM proteins or deconvolute whether the interactions occur with the native, full‐length gene product or bioactive fragments.

### Predicting interactions using protein sequence analysis

Protein–protein interactions can be predicted using sequence, domain, and 3D structure analyses using different online resources (Table 5), including some dedicated to ECM proteins (Table 6). These analyses use models trained on experimental datasets such as those of the RCSB PDB [40] or the 3did database comprised of three‐dimensional interacting domains and domain‐based structural interaction templates [57] including domains commonly found in ECM proteins, such as FN type I, II, and III, epidermal growth factor (EGF) and EGF‐like, von Willebrand A and thrombospondin‐1 domains [1, 2, 31]. Domain–domain interactions can be inferred computationally using Protein–Protein Interactions Domain Miner (PPDM) based on manually curated databases of physical interactions, predicted interactions, and complex structures [58]. Docking methods based on 3D structures can be used to predict biomolecular interactions and characterize binding interfaces [73]. Among these, the algorithm AutoDock Vina 1.2.0 allows the simultaneous docking of multiple ligands and a batch mode for docking a large number of ligands, including drugs [74]. Of relevance to ECM research, this algorithm was used to show that three biomarkers of colon adenocarcinoma, a core matrisome protein, procollagen C‐proteinase enhancer‐2, and two matrisome‐associated proteins, leptin and nerve growth factor, could bind to the tumor suppressor dictamnine [75].

**Table 5:** Computational tools for the prediction and visualization of protein interactions.

Resource	Description	Link	References
The Proteomics Standard Initiative Common QUery InterfaCe (PSICQUIC)	Standardized website for querying molecular interactions across a wide range of interaction databases	http://www.ebi.ac.uk/Tools/webservices/psicquic/view/home.xhtml	Del‐Toro *et al*., 2013 [149]
Cytoscape	Open‐source software platform for visualizing complex networks and integrating them with any type of attribute data. Connected to interaction databases. Numerous apps for network analysis (functional enrichment and network statistics)	https://cytoscape.org/	Shannon *et al*., 2003 [151]
The Reactome Pathway Database	Manually curated database for querying biological processes in normal and disease states through pathway browsers and mapping, over‐representation, and expression analysis tools. Allows for the discovery of functional relationships through ‐omics data	https://reactome.org/	Milacic *et al*., 2024 [153]
Metascape	Web‐based portal designed to provide a comprehensive gene list annotation and analysis resource. Integrates over 40 independent knowledge bases	https://metascape.org/	Zhou *et al*., 2019 [154]
Interactome3D	Web service for structural annotation and modeling of protein–protein interaction networks	https://interactome3d.irbbarcelona.org/	Mosca *et al*., 2013 [57]
IntAct (IMEx Consortium member)	Built‐in network visualization tool to visualize binding sites and the effect of mutations on interactions. The tool allows you to keep or discard Spoke's expanded interactions	https://www.ebi.ac.uk/intact/home	Del Toro *et al*., 2022 [50]
STRING	Provides network statistics and functional enrichment analysis	https://string‐db.org/	Szklarczyk *et al*., 2023 [55]
AutoDock Vina	Open‐source program for simulating molecular docking	https://vina.scripps.edu/	Eberhardt *et al*., 2021 [74]
AlphaFold 3	Tool for the prediction of the structure of complexes, including proteins, nucleic acids, small molecules, ions, and modified residues	https://alphafoldserver.com/welcome	Abramson *et al*., 2024 [76]
RoseTTAFold All‐Atom	Biomolecular structure prediction tool that integrates protein, nucleic acid, and small molecule representations using atom‐bond graphs and multi‐scale input features (1D, 2D, 3D) to iteratively generate physically possible molecular complexes	https://github.com/baker‐laboratory/RoseTTAFold‐All‐Atom	Krishna *et al*., 2024 [77]

**Table 6:** Specialized computational tools for the prediction and visualization of ECM protein interactions.

Resource	Description	Link	References
The ECM interaction database MatrixDB	Includes a Network Explorer to generate context‐specific (tissue‐ and disease‐specific) PPI networks based on transcriptomic data from Bgee and ECM proteomic data from MatrisomeDB	https://matrixdb.univ‐lyon1.fr/	Samarasinghe *et al*., 2025 [62]
MatriCom	scRNA‐Seq data mining tool to infer ECM‐ECM and cell‐ECM communication systems. It relies on a unique database, MatriComDB, that includes curated interactions involving matrisome components	https://matrinet.shinyapps.io/matricom	Lamba *et al*., 2025 [69]
MatriNet	Database for studying the structural changes in ECM network architectures as a function of their protein–protein interaction strengths across 20 different tumor types	https://github.com/MatriNet/matrinetR	Kontio *et al*., 2022 [150]

Methods based on deep learning, such as AlphaFold 3 [76] and RoseTTAFold All‐Atom [77], use protein sequences and ligand‐specific feature representations as inputs and predict the structure of complexes comprised of proteins and a variety of partners, from ions and chemicals to nucleic acids, thus predicting biomolecular interactions. A newly developed tool, Combfold [78], uses a combinatorial assembly algorithm and AlphaFold2 to predict structures of large protein complexes. However, so far, these methods have not been benchmarked on the matrisome, and their impact on the discovery of novel ECM interactions has not been evaluated. In addition, the building of training sets of ECM protein interactions would improve the robustness of the prediction of ECM protein interactions.

## Experimental approaches to identify partners and interactors of ECM proteins

### Biophysical approaches to characterize ECM protein–protein interactions

#### Biophysical approaches requiring protein immobilization

**Fig. 3:**
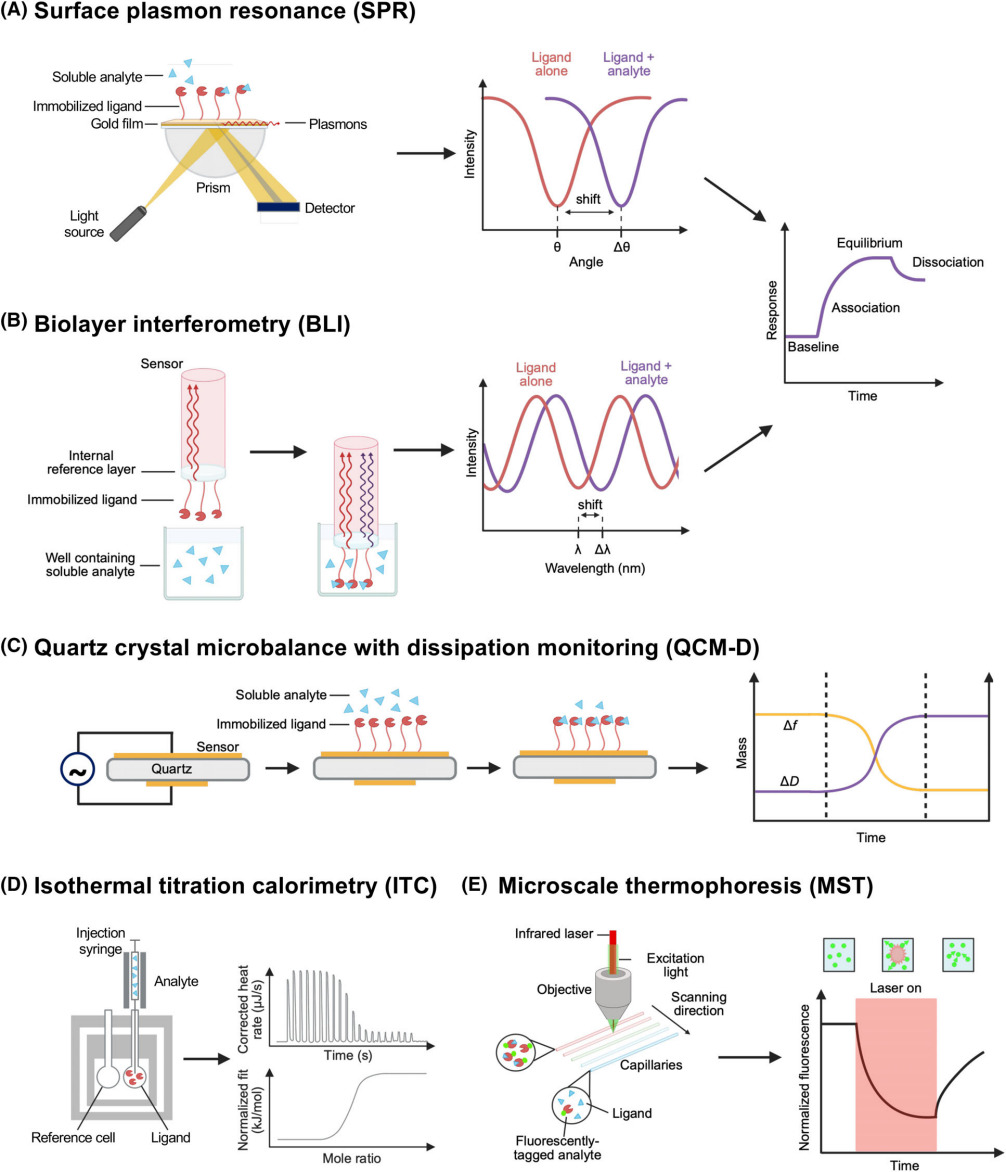
Biophysical methods to characterize ECM protein interactions. (A) Surface plasmon resonance (SPR). A ligand (red) is immobilized onto a gold film covered with carboxymethyl dextran, and the analyte (blue) is flowed via a microfluidic cartridge over the sensor surface. The SPR signal, expressed in resonance units (RU), is measured as a function of time, and the resulting curve is called a sensorgram. The SPR principle is described in the text, and the parameters measured by this technique are listed in Table 7. (B) Biolayer interferometry (BLI). A ligand (red) is immobilized on the end of a sensor tip that is dipped into a solution containing the putative binding partner or analyte (blue). The BLI signal is recorded as a function of time. The BLI principle is described in the text and the parameters measured by this technique are listed in Table 7. (C) Quartz crystal microbalance with dissipation (QCM‐D) monitoring. Voltage is applied to a quartz crystal that has electrodes on either side, causing the crystal to oscillate at a specific resonance. The resonance frequency (*f*) relates to the mass of the quartz disk. When a ligand is immobilized onto a sensor surface and the analyte is introduced, the thickness of the quartz changes, resulting in a change in frequency (Δ*f*). Dissipation (Δ*D*), or the loss of energy due to deformation such as binding, can also be determined. The QCM‐D principle is described in the text and the parameters measured by this technique are listed in Table 7. (D) Isothermal titration calorimetry (ITC). A protein in solution is titrated with the other protein in solution. Binding is measured by the change in heat of the sample solution in comparison to the reference cell (typically water). The thermodynamics of the interaction can be quantified as the change in heat over time, as well as the mole ratio of the interaction. The ITC principle is described in the text and the parameters measured by this technique are listed in Table 7. (E) Microscale thermophoresis (MST). Various concentrations of fluorescently tagged or intrinsically fluorescent ligand (red) are mixed with non‐fluorescent putative interactants (blue) in capillaries. The MST principle is described in the text, and the parameters measured by this technique are listed in Table 7. This figure was created in https://BioRender.com.

Common biophysical techniques to characterize ECM PPIs include optical techniques such as surface plasmon resonance (SPR) and biolayer interferometry (BLI), an acoustic technique, quartz crystal microbalance with dissipation monitoring (QCM‐D), or, although less frequently used, optical tweezers. These techniques are performed with one partner (termed “ligand”; Box 1) covalently immobilized or captured by affinity on a solid surface and one partner (termed “analyte”; Box 1) in solution. In brief, SPR assays are performed by injecting an analyte in a buffer flow over a ligand immobilized on a sensor chip. Binding events are detected as a change in mass at the sensor surface that is proportional to the changes in refractive index occurring above the sensor chip surface. This resonance angle shift is recorded as a sensorgram with the response measured in resonance units (RU) monitored over time (Fig. 3A) [79, 80]. In the context of ECM proteins, SPR has, for example, been used to characterize interactions of core matrisome proteins, including laminins [81], nidogen‐2 [82], and fibrillin microfibrils and elastic fiber proteins [83].

Box 1 **Terminology**

Interaction participants:

- The protein of interest is referred to as the immobilized ligand in biophysical methods and as the bait in biochemical methods.
- The partners of a given protein of interest are referred to as analytes in biophysical methods and as preys in biochemical methods. Importantly, the partners may or may not directly bind to the protein of interest (see below).

Definitions of the controlled vocabulary “Molecular Interactions” available in the Ontology Lookup Service https://www.ebi.ac.uk/ols4/:

- Association: Interaction between molecules that may participate in the formation of one, but possibly more, physical complexes. Often describes a set of molecules that are co‐purified in a single pull‐down or co‐immunoprecipitation, but might participate in the formation of distinct physical complexes sharing a common bait.
- Physical interaction: Interaction between molecules within the same physical complex. Often identified under conditions that suggest that the molecules are in close proximity but not necessarily in direct contact.
- Direct interaction: Interaction between molecules that are in direct contact with each other.
- Binary interaction: One bait/one prey.
- n‐ary interaction: One bait/n preys.
- Spoke expansion: Complex n‐ary data can be deconvoluted into binary interactions using the spoke expansion model. This model assumes that all molecules within a complex interact with a single designated molecule, usually the bait.

On the other hand, BLI experiments are performed with a ligand immobilized on a sensor tip, which is then dipped into the analyte solution. The binding of the analyte to the sensor surface induces a change in the thickness of the biolayer at the sensor surface, which is measured by changes in the interference pattern of white light reflected from two surfaces: a layer of the immobilized ligand on the sensor tip and an internal reference layer (Fig. 3B) [84]. BLI has been used to study the interactions of matrisome proteins or protein domains, including the propeptide of lysyl oxidase and lysyl oxidase‐like 2 [85, 86], or integrins [87].

QCM‐D measures changes in mass at the sensor surface upon binding of an analyte to a ligand immobilized on the surface of a sensor (Fig. 3C) [88, 89, 90]. Importantly, if performed with purified proteins, all these techniques can provide information on direct binary interactions. QCM‐D has been applied to study the mechanisms of cell adhesion and has identified interactions between integrin and the adhesive RGD peptide during the early stages of cell adhesion [91], and between human ovarian cancer cells and FN and vitronectin [92].

Optical tweezers, a single‐molecule technique in contrast to the biophysical approaches described above, use a laser to exert a force on a dielectric bead and optically trap the bead at a controllable position in three dimensions. It can be used to apply and measure mechanical forces in molecular systems and to investigate biomolecular interactions when biomolecules (*e.g*., proteins) are bound to beads. Kinetics, affinity, and energy parameters (the transition state ΔG≠, the distance to the transition state Δx≠) of interactions can be determined depending on the experimental setup [93]. This method is not frequently used to investigate ECM PPIs, but it has provided insights into the interaction of cells with the ECM receptors. For example, optical tweezers experiments have shown that syndecan‐4, a membrane proteoglycan, increased the lifetime of the binary Thy‐1‐αvβ3 integrin complex by interacting directly with Thy‐1 [94]. Optical tweezers have also been used to measure the rupture force between fibrinogen and either purified αIIbβ3 integrin or αIIbβ3 integrin on platelets [95], between fibroblasts and fibronectin [96] and to investigate the mechanisms and kinetics of fibroblast adhesion to fibronectin [97].

#### Biophysical approaches using ligands and analytes in solution

In contrast to the methods described above, techniques like isothermal titration calorimetry (ITC) and microscale thermophoresis (MST) are performed with both partners in solution. ITC is performed at constant temperature and measures the heat change that occurs when two molecules interact during the titration of the ligand into the sample cell containing the other interactant (Fig. 3D) [98, 99, 100]. ITC has been used to characterize the molecular basis of the binding of the laminin N‐terminal domain with Ca^2+^ [101] and of collagen I self‐assembly [102]. In MST assays, a fluorescently labeled protein is mixed with various concentrations of a non‐fluorescent ligand. The samples are then loaded on capillaries and submitted to a temperature gradient. The movement of the fluorescent protein in the temperature gradient and the effect of the different ligand concentrations are recorded and provide binding information (Fig. 3E) [99]. MST has been applied to determine the affinity of integrins α3β1, α5β1, α6β1, and peptide ligands [103].

Different parameters characterizing protein–protein interactions can be obtained from these techniques (Table 7). Since SPR, BLI, MST, and QCM‐D monitor biomolecular interactions in real time, they provide values of kinetic parameters (*i.e*., the association rate, governing molecular recognition, and the dissociation rate, indicative of the stability of the interactions), in addition to the equilibrium dissociation constant (KD), which reflects the binding affinity and interaction strength. The dissociation rate can be used to estimate the half‐life of PPIs. While we will focus here on describing the application of these techniques to probe PPIs, they can also be used to study protein interactions with GAGs, nucleic acids, ions, chemicals, or drugs. Readers interested in learning more about these techniques can refer to the website of the Center for Molecular Interactions at Harvard Medical School (https://cmi.hms.harvard.edu/technologies) or the Biolin Scientific website for QCM‐D (https://www.biolinscientific.com/measurements/qcm‐d#how‐does‐qcm‐d‐work).

**Table 7:** Major characteristics of common biophysical techniques used to profile ECM protein interactions.

	Surface plasmon resonance (SPR)	Biolayer interferometry (BLI)	Isothermal titration calorimetry (ITC)	Quartz crystal microbalance with dissipation monitoring (QCM‐D)	Microscale thermophoresis (MST)
Label‐free method	+	+	+	+	+ *only for intrinsically fluorescent proteins*
Method involving the immobilization of one partner	+	+	–	+	–
Immobilization‐free method	–	–	+	–	+
Determination of binding affinity	+	+	+	+	+
Determination of binding kinetics	+	+	–	+	+
Calculation of thermodynamic parameters	+	–	+	–	+
Detection of conformational changes	–	–	–	+	–
Coupling with mass spectrometry	+	+	–	+	–

#### High‐throughput approaches

Although these techniques are primarily used to identify and characterize binary interactions or a few interactions simultaneously with purified proteins, they can also be adapted to a multiplexed array format where hundreds of interactions can be monitored simultaneously using SPR imaging (SPRi) or by fluorescence. For example, arrays comprised of more than 8200 proteins and fluorescence detection have been used to identify proteins interacting with GAGs and the proteoglycan osteoglycin [104]. ECM arrays probed by SPRi to screen about 130 interactions resulted in the discovery of novel partners of endostatin, a proteolytic fragment of collagen XVIII [105], of the core matrisome protein, procollagen C‐proteinase enhancer‐1 [106], and of the matrisome‐associated protein, the ECM regulator lysyl oxidase‐like 2 [86, 107]. Liquid chromatography coupled to tandem mass spectrometry (LC–MS/MS) can also be used to identify potential partners of an immobilized ligand captured using SPR or BLI from complex biological media (*e.g*., serum, cell lysate, or subcellular fractions) [108, 109]. Multiparametric SPR using multiple wavelengths of light provides information on surface coverage and the biolayer thickness, allowing sample conformation analysis during binding events. This approach has, for example, been used to design an MMP‐9 sensor based on immobilized synthetic peptides as substrates. Upon MMP‐9 binding, the peptides are hydrolyzed, resulting in a decrease in the SPR signal [110].

#### Limitations

While powerful, all these techniques present some limitations. First, in their standard design, they require a list of candidate partners to be tested and purified proteins to be available. In addition, the immobilization of a protein on a sensor surface for SPR or BLI assays may alter its conformation and/or restrict or prevent access to its potential binding sites. Although they can be performed using protein intrinsic fluorescence, MST experiments typically require the conjugation of the protein of interest with a fluorophore, which could alter its conformation and, hence, its ability to bind to its partners.

### Classical biochemical approaches to identify partners of ECM proteins

Biochemical approaches, such as pull‐down and co‐immunoprecipitation, are methods that have been broadly employed to investigate interactions between two proteins, often in a targeted, that is, hypothesis‐driven, manner. Using these methods, it is possible to identify partners of a protein of interest with specificity or sensitivity, albeit in a low‐throughput manner compared to higher‐throughput methods that will be discussed below (Table 8).

#### Pull‐down assay

**Fig. 4:**
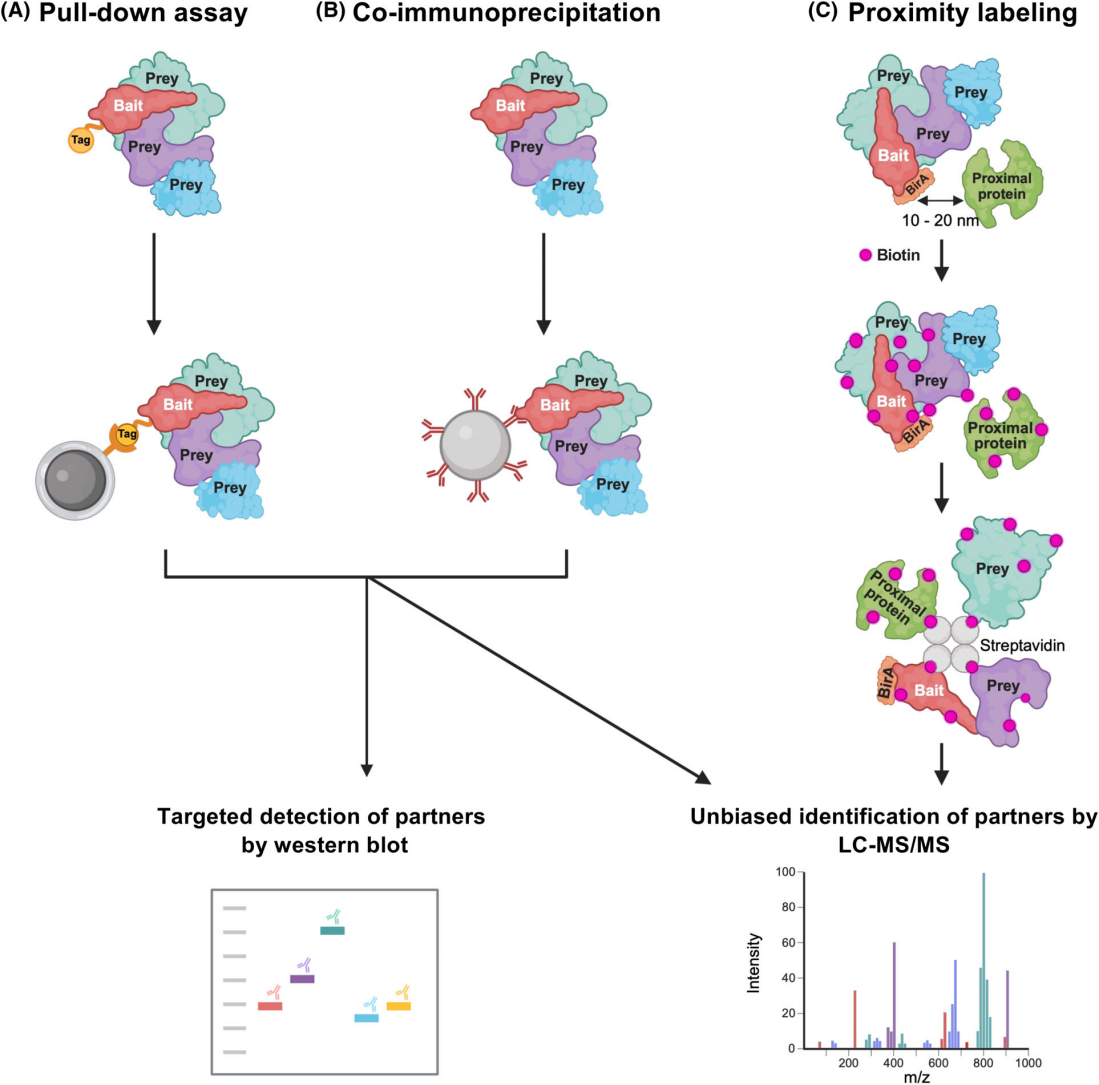
Biochemical methods to identify partners and interactors of ECM proteins. Schematic representation of biochemical methods to identify direct (purple and teal) and indirect (blue) interacting partners (preys) of a protein of interest (bait, red). (A, B) Affinity purification‐based techniques include pull‐down assay that captures a tagged bait protein and its partners (A) and co‐immunoprecipitation assay (co‐IP) that captures a bait and interacting partners using an antibody recognizing the bait or its tag (B). The protein preys can be identified by western blotting or by an unbiased, high‐throughput manner using LC–MS/MS. (C) In proximity labeling coupled to mass spectrometry (PL‐MS), an enzyme‐tagged bait protein (red) biotinylates proteins located within ~ 10–20 nm. Biotinylated proteins are pulled down using streptavidin beads and digested into peptides. Peptides can then be identified via LC–MS/MS. This figure was created in https://BioRender.com.

Pull‐down assays require the capture of a tagged protein of interest (termed the “bait”) on a solid surface (*e.g*., beads or resin) via affinity. A biological sample containing a tagged bait and potential interactants (termed “preys”) is incubated with an affinity surface to immobilize the bait, and interactants that are “pulled down” together with the bait can be identified in a targeted manner by western blot or in an unbiased manner using LC–MS/MS (Fig. 4A). The bait can be purified or captured from complex biological media, such as a lysate of cells overexpressing the tagged bait. Practically, a pull‐down assay requires basic laboratory skills and is relatively easy to set up in terms of training, equipment, and cost, which makes this approach appealing [111].

Multiple examples from the literature demonstrate the relevance of this method to probe ECM PPIs. For instance, Fogelgren *et al*. used pull‐down assays to validate the interactions between LOX and fibronectin [112] initially identified by yeast 2‐hybrid (Y2H), another molecular approach to identify protein–protein interactions in yeast cells [113]. Pull‐down assays were also used to validate the interactions identified by Y2H between ADAMTS‐1 and fibulin 1 [114] or between protein‐1 (BMP‐1) and fibronectin [18].

#### Co‐immunoprecipitation

Co‐immunoprecipitation (co‐IP) is similar to a pull‐down assay, but the bait is immobilized by an anti‐bait or anti‐tag (*e.g*., FLAG, His) antibody coupled to a solid surface (*e.g*., beads or resin) (Fig. 4B). This method is well established to test interactions between native proteins and, similarly to pull‐down assays, is relatively accessible and easily adaptable by researchers [111]. We used co‐IP to confirm the interaction, initially identified by SPRi, between procollagen C‐proteinase enhancer‐1 and endostatin [106]. We have also used co‐IP to validate the interactions between the N‐propeptide of the α1 chain of collagen V and several ECM proteins, including fibronectin, tenascin C, the Tissue Inhibitor of Metalloproteinase 1 (TIMP1), and TGFβ1, initially identified by Y2H [115].

#### Limitations

Pull‐down and co‐IP assays present similar limitations. First, unless a pull‐down is performed with two purified proteins, these assays cannot distinguish whether the preys identified interact directly with the bait or whether the bait and preys are part of the same molecular complex and their apparent bait–prey interaction is mediated by a third molecule binding to both (Fig. 4A,B). In addition, adding an affinity tag to a protein and/or capturing it on a solid surface may affect its conformation and ability to bind to other proteins. Commonly used agarose beads have more porous surfaces than magnetic beads, which can lead to increased nonspecific binding, especially when attempting to perform experiments with ECM proteins that are heavily glycosylated and hence quite sticky. In addition, neither method permits the detection of low‐affinity or transient interactions. These assays also cannot provide information on the kinetics or affinity of the interactions detected. Lastly, these assays can only detect interactions between soluble proteins and cannot resolve PPIs within the insoluble assembled ECM scaffold.

### Mass‐spectrometry‐based approaches for high‐throughput interactomics

Unbiased and high‐throughput methods such as affinity purification and proximity labeling coupled with MS (AP‐MS and PL‐MS, respectively) have emerged as powerful tools to comprehensively characterize and map protein interactomes at a proteome scale [116, 117] (Table 8).

#### Affinity purification coupled with mass spectrometry

AP‐MS is a widely employed proteomic technique to purify protein complexes and characterize their composition (Fig. 4A,B). This method involves the selective enrichment of a target protein, the bait, along with its partners present in the biological sample being tested. Following IP or pull‐down, the proteins that co‐purify with the bait are subjected to on‐bead proteolytic or in‐solution digestion after elution from the affinity matrix. The resulting peptides are then analyzed by LC–MS/MS [116, 118] (Fig. 4A,B), allowing for the unbiased identification of proteins (preys) associated with a bait in the same complex(es). However, in AP‐MS experiments, a single immobilized bait captures n preys (*i.e*., n potential binding partners) and does not allow the experimental identification of binary interactions within complexes. Complexes are thus represented in databases as binary interactions, usually using spoke expansion (Box 1), assuming that the bait interacts with each prey, which does not necessarily reflect what happens *in vivo*.

AP‐MS has been applied to probe the ECM PPIs. For example, Mahdy and colleagues used AP‐MS to decipher the interactome of the ECM‐affiliated protein annexin A2 in lysates of MDA‐MB‐231 cells. They identified known annexin A2 partners (e.g., receptor for activated C kinase 1 and the AHNAK nucleoprotein) and new partners belonging to the matrisome, such as cellular communication network factor 1 (CCN1 or Cyr61), fibronectin, and thrombospondin‐1 [119]. The intracellular proteostasis network of collagen I was investigated by tagging COL1A1 and COL1A2 with hemagglutinin (HA) and FLAG tags, respectively, in HT‐1080 fibrosarcoma cells [120]. Following chemical crosslinking to stabilize transient or weak protein–protein interactions, AP‐MS was performed using antibodies against the HA and FLAG tags. The analysis identified known components of the collagen proteostasis network, such as heat shock protein 47, peptidylprolyl isomerase B (PPIB), FK506 binding protein 65 (FKBP65), and protein disulfide isomerase (PDI), as well as new interactors, including aspartyl/asparaginyl β‐hydroxylase (ASPH) and the β‐subunit of glucosidase II [120]. This study further revealed the functional importance of these new interactions by showing that ASPH was required to hydroxylate an aspartic acid residue in the COL1A1 N‐propeptide, a previously unidentified posttranslational modification.

Of note, AP‐MS relies on the capture of proteins, which can only be effectively achieved using soluble or solubilized proteins. This condition is particularly well suited to study intracellular protein complexes. However, the solubilization conditions typically employed for AP‐MS are inappropriate to solubilize ECM protein supramolecular assemblies [121], limiting the applicability of this technique to study the native ECM protein interactions.

#### Proximity labeling coupled to mass spectrometry

To overcome some of the limitations associated with affinity purification, such as the requirement for proteins to be soluble, proximity labeling (PL) has emerged as a complementary approach that enables the spatial mapping of protein interactions [122, 123]. In this approach, a bait protein is genetically fused to biotin ligases (*e.g*., BioID, BioID2, TurboID) or peroxidases (*e.g*., APEX) that catalyze the generation of reactive biotin species [122]. These activated intermediates covalently label nearby proteins *in situ* (within ~ 10–20 nm), typically on lysine (BioID) or tyrosine (APEX) residues. Biotinylated proteins are subsequently solubilized, enriched using streptavidin‐based affinity capture, digested into peptides, and identified via LC–MS/MS [122] (Fig. 4C). The covalent nature of biotin labeling, which resists harsh lysis and wash conditions, enables the detection of weak or transient interactions and is resistant. Recent advances have enabled the modulation of labeling radii (Table 8) and kinetics, allowing for both spatial and temporal resolution of protein interactions [116, 122, 124].

**Table 8:** Major characteristics of the main biochemical and imaging techniques used to identify ECM protein interactions. ‐

	Pull‐down assay	Co‐immuno‐precipitation (co‐IP)	Affinity purification coupled to mass spectrometry (AP‐MS)	Proximity labeling coupled to mass spectrometry (PL‐MS)	Förster resonance energy transfer (FRET)	Proximity ligation assay (PLA)
Labeling/tagging required	+	–	+	+	+	+
Labelling radius	–	–	–	10–20 nm	< 10 nm	< 40 nm
Identification of direct binding partners	+	+	+	–	–	–
Identification of proximal proteins	–	–	–	+	+	+
Coupling with LC–MS/MS	–	–	+	+	–	–
*In‐situ* capability (labeling and/or detection)	–	–	+ Labelling only	–	+ Labelling and detection	+ Labelling and detection

Using PL‐MS, Peeney *et al*. reported the profiling of the extracellular interactome of tissue inhibitor of metalloproteinase 2 (TIMP2) [125]. To do so, they fused BioID2 or TurboID to TIMP2 to label the proteins in proximity to TIMP2. Enrichment of biotinylated proteins and subsequent LC–MS/MS analysis identified known interactors of TIMP2, such as MMP‐2, but also novel candidate partners, such as cellular communication network factors CCN1 and CCN2 (also known as CYR61 and CTGF), and thrombospondin‐1. Furthermore, this study reported that the binding of MMP14 to MMP2 is mediated by the N terminus of TIMP2, showing that PL can be used to identify specific domain(s) involved in protein interactions. The application of PL to a 3D spheroid model led to the identification of additional extracellular interactants of TIMP2, such as latent TGF‐β binding protein 3, complement component C3, acidic chitinase, and Rac family small GTPase 2. More recently, Dusadeemeelap and colleagues aimed to identify potential interactors of phosphate‐regulating endopeptidase X‐linked (PHEX) in osteoblasts. Indeed, although the role of PHEX in regulating the mineralization of bone is well known, the knowledge of its interacting partners is limited. Using BioID2 fused to the catalytically active C‐terminal extracellular domain of PHEX, they identified 39 proteins proximal to PHEX, including several matrisome components (periostin, biglycan, thrombospondin‐1, fibronectin, the α1 chain of collagen I, and the α1, α2, and α3 chains of collagen VI) [126]. Co‐IP assays were further performed and confirmed direct interactions between PHEX and periostin and biglycan [126]. Of note, PL‐MS has been used to characterize the integrin interactome, also termed the adhesome [127, 128, 129].

While very powerful and now broadly adopted, PL‐MS presents some limitations. This method is designed to identify proteins in proximity to a protein of interest rather than directly interacting proteins. BioID‐based labeling is inefficient for ECM components such as collagens, which undergo post‐translational lysine hydroxylation [1], rendering some lysine residues less prone to biotinylation. Additionally, the large size and multimeric nature of many ECM proteins complicate both labeling and downstream analysis. Fusion of enzymes to ECM proteins may disrupt their native conformation, intracellular trafficking, or extracellular assembly. Last, care should be applied to maximize the identification of ECM peptides and proteins by MS. This includes allowing ECM‐specific posttranslational modifications, including hydroxylation of proline and lysine residues, as variable modifications during the database search [130, 131].

### Visualization of protein–protein interactions

Despite advances in interactome mapping, most studies of ECM interactions have focused on purified proteins or soluble protein extracts, which fail to capture the native spatial and structural organization of the ECM. This limitation conceals critical insights into how molecular interactions are organized and regulated *in situ*. Techniques such as Förster resonance energy transfer (FRET) and proximity ligation assay (PLA) offer solutions by enabling the visualization of protein in close proximity (10^−8^ m resolution) within native ECM environments (Table 8). These techniques thus add an important spatial dimension to ECM interactome studies that is inaccessible through bulk analyses. However, as discussed above, it is important to keep in mind that two proteins in close proximity may not interact directly.

#### Förster resonance energy transfer

**Fig. 5:**
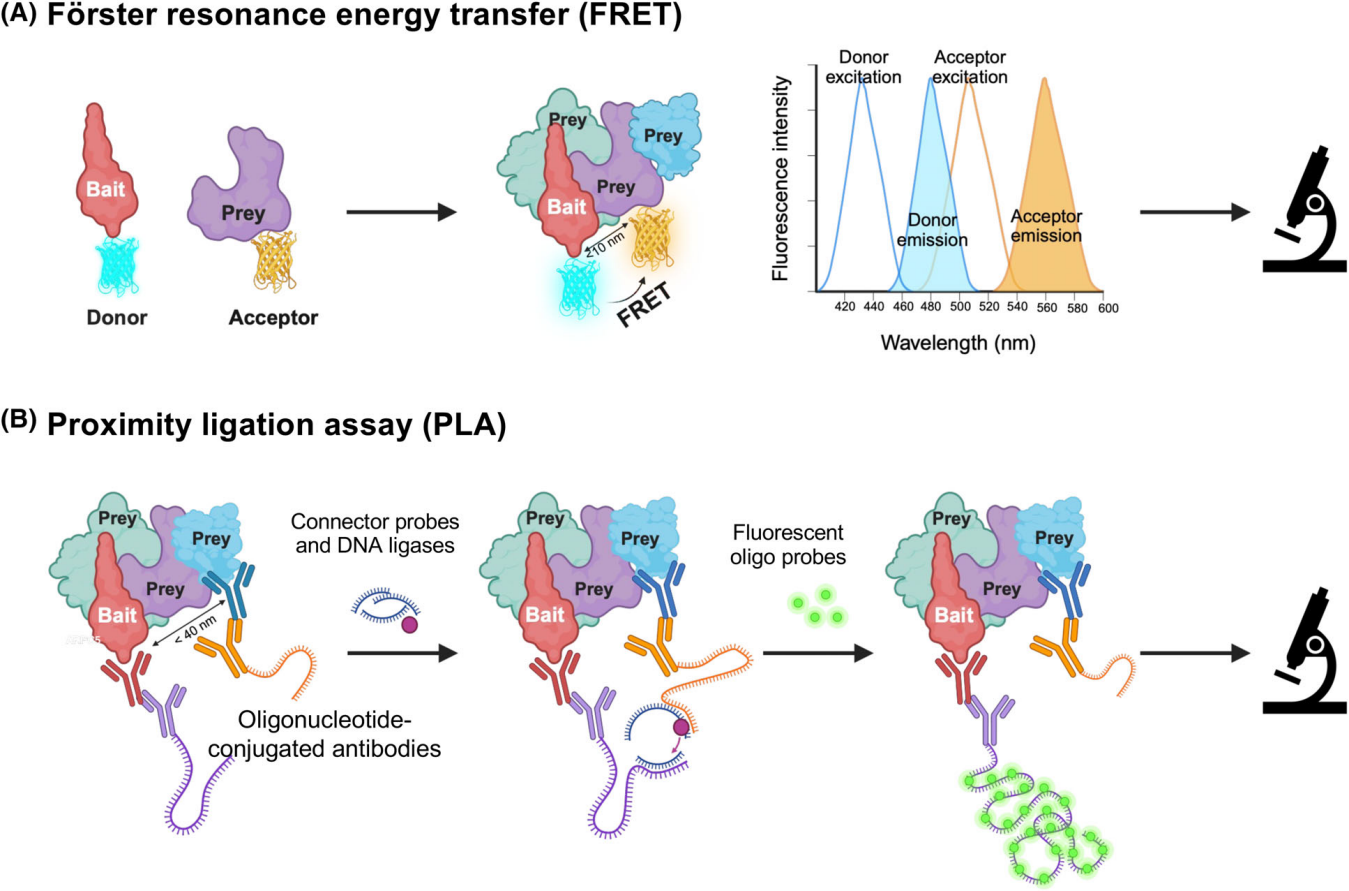
Methods to identify partners and interactors of ECM proteins. (A) Förster resonance energy transfer (FRET) enables the visualization of protein interactions *in situ* by detecting the energy transfer between a donor fluorophore (blue) and an acceptor fluorophore (yellow). The energy transfer occurs when the protein fused to the donor fluorophore (red) and the protein fused to the acceptor fluorophore (purple) are within 10 nm of each other. (B) Proximity ligation assay (PLA) uses antibody‐conjugated oligonucleotides to detect a protein (blue) found in close proximity to a protein of interest (red). This method uses rolling circle amplification to produce a fluorescent signal when both proteins are within 40 nm of each other. This figure was created in https://BioRender.com.

FRET is a distance‐dependent molecular interaction wherein energy is non‐radiatively transferred from an excited donor fluorophore to a nearby acceptor fluorophore [132, 133, 134, 135] (Fig. 5A). This process occurs when the donor and acceptor are within a Förster distance of typically ≤ 10 nm. Common strategies for protein labeling include chemical conjugation to amine or sulfhydryl groups or genetic fusion to fluorescent tags. Because of its ability to detect nanometer‐scale changes, FRET has become a state‐of‐the‐art approach to studying conformational changes, structural dynamics, and *in‐ situ* protein–protein interactions in real‐time and native environments [134].

FRET has been widely applied to investigate molecular interactions in focal adhesions, particularly those involving integrins and associated cytoskeletal proteins [136, 137, 138, 139]. More recently, FRET has been extended to study ECM protein interactions. FRET was used to examine the mechanical regulation of transglutaminase 2 (TG2) binding to FN fibers [140]. In this study, primary amines of denatured FN dimers were labeled with Alexa Fluor 488 (donor), and the four cysteines in the FNIII7 and FNIII15 modules, which are otherwise cryptic in native FN, were labeled with Alexa Fluor 546 (acceptor). Under relaxed conditions, the proximity of the donor and acceptor within the FN fibers resulted in a high FRET signal. In contrast, mechanical stretching of the FN fibers led to a lower FRET signal. These observations demonstrated that FRET can effectively report the strain‐dependent conformational changes in FN fibers previously reported [141]. The study further tested the preferential binding of different conformations of TG, labeled with Alexa Fluor 647, to stretched or relaxed FN fibers by measuring the TG2‐AF647 fluorescence intensity normalized to the excited FN‐FRET intensity, and found that TG2 in its closed conformation exhibited preferential binding to relaxed FN fibers, while TG2, in its open conformation, retained binding regardless of FN tension [140]. This example underscores FRET's utility in studying protein interactions and conformational dynamics under physiologically relevant mechanical conditions.

It is worth noting that amine or sulfhydryl‐based labeling presents limitations, including non‐site‐specific modification. Alternative approaches include indirect immunolabeling using fluorophore‐conjugated antibodies, which, while enabling specific targeting, can reduce FRET efficiency due to increased donor–acceptor distances [132]. Genetically encoded fluorescent protein fusions offer another strategy that has been widely used to study focal adhesions [137]. However, overexpression of fusion constructs may perturb protein localization and function, and the tags themselves can induce steric or conformational effects that might interfere with native protein interactions.

#### Proximity ligation assay

Proximity ligation assay (PLA) overcomes limitations associated with chemical labeling in FRET and can complement FRET analyses by allowing highly specific and sensitive detection of endogenous protein interactions in fixed samples, preserving their spatial organization. In brief, this method employs two primary antibodies raised in different species, each targeting one of the two proteins of interest. Secondary antibodies, conjugated to unique oligonucleotides, bind to the Fc regions of the primary antibodies (Fig. 5B). When the target proteins are in close proximity (< 40 nm; Table 8), the conjugated oligonucleotides serve as a template for ligation and rolling circle amplification, which, in the presence of polymerases and fluorescently labeled oligonucleotides, generates a detectable fluorescent signal observable by microscopy [142].

PLA has been used to investigate different aspects of ECM biology. Using a multiplex bead‐based immunoassay on 148 osteoarthritic cartilage samples, Danalache and colleagues showed that MMP‐2 and MMP‐3 mediate the degradation of collagen VI, while MMP‐3 and MMP‐7 can digest perlecan [143]. Using PLA, they further confirmed the proximity, in osteoarthritic cartilage, of MMP‐2 with collagen VI, MMP‐7 with perlecan, and MMP‐3 with collagen VI and perlecan [143]. PLA was also used to investigate the role of MMP‐2 and MMP‐9 in the processing of dentin sialophosphoprotein (DSPP) and found that MMP‐2 and MMP‐9 localized in close proximity to dentin sialoprotein in murine molar odontoblasts [144]. PLA is also widely used to visualize *in situ* PPIs identified by other techniques. For example, thrombomodulin, an endothelial cell surface glycoprotein, was shown to bind fibronectin selectively through its lectin‐like domain *in vitro* using a solid‐phase binding assay [145]. PLA confirmed the colocalization of the two partners in the melanoma vasculature *in vivo*. Interestingly, perinuclear PLA signals were observed, suggesting potential intracellular co‐trafficking or co‐recycling events [145]. PLA also identified MMP‐14, CCN1, and CCN2 as being in close proximity to, and hence as being potential binding partners, of TIMP2 [125]. PLA has also been used to probe the integrin adhesome [146].

PLA is thus well suited to visualize PPIs within cells or intact tissues in the context of a native ECM organization and can provide valuable spatial information on these PPIs. However, the technique relies on the availability of highly specific antibodies for each protein of interest. Adequate negative controls are essential to minimize false positives due to nonspecific antibody binding [147]. Additionally, due to its proximity requirement (40 nm), which is lower than other interaction assays (Table 8), observations from PLA should be cross‐validated using complementary techniques such as multiplexed imaging and super‐resolution microscopy to enhance spatial resolution [148]. Last, PLA has an inherently low throughput and provides potential binary interaction data, making it more suited for targeted validation than comprehensive interactome profiling.

## A roadmap to building ECM interaction networks

**Fig. 6:**
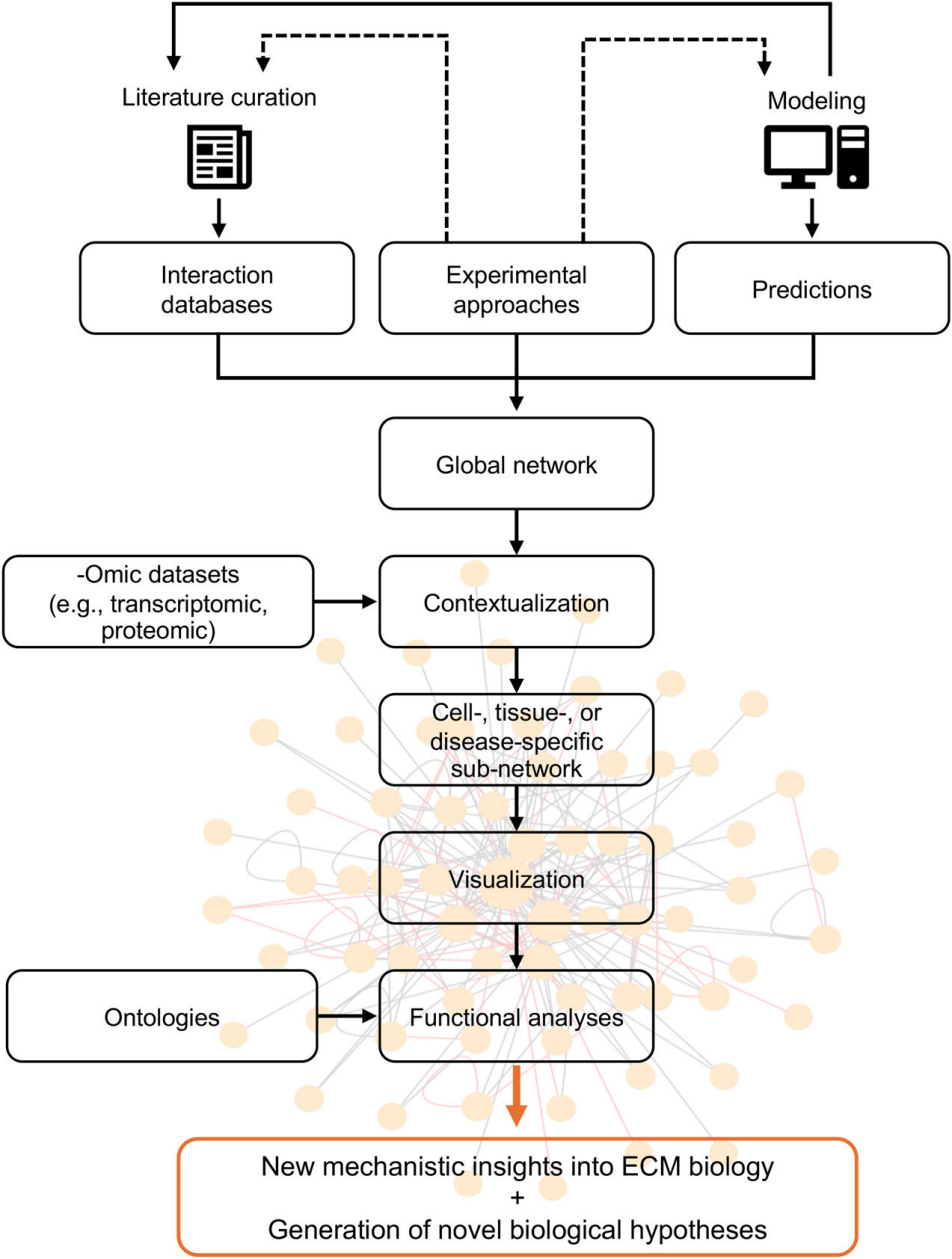
A roadmap to building the interaction network of ECM proteins. Schematic representation of the different steps that researchers can undertake to construct the interaction network of a protein of interest.

To guide readers interested in gaining insights into the interactome of an ECM protein of interest, we propose here a roadmap based on a three‐pronged approach combining (a) the query of curated PPI databases such as the Proteomics Standard Initiative Common Query Interface (PSICQUIC, [149]), (b) the prediction of interactions using molecular modeling algorithms (of note, some of these predictions are already included in certain PPI databases), and (c) the design and performance of biophysical and biochemical experiments to generate a global interaction network of a protein of interest (Fig. 6). Tables 5 and 6 lists recommended tools to apply this roadmap. Since the composition and stoichiometry of multimeric complexes are context‐dependent (*e.g*., cell‐ and tissue‐level localization, pathophysiological state) and vary as a function of protein abundance and the proteoforms involved, we encourage researchers to factor in these elements to build biologically relevant interaction networks. The global network obtained can further be contextualized by integrating transcriptomic data on gene expression or proteomic data on protein presence and abundance. Contextualization is achieved by filtering out the genes or proteins of the global network not expressed or detected in the “context” of interest, for example, the tissue or disease of interest. Network contextualization thus results in the generation of tissue‐ or disease‐specific subnetworks and can leverage data from MatrisomeDB [44] or MatriNet [150]. Both global and subnetworks can then be visualized using various tools, the most popular being the open‐source software platform Cytoscape [151]. Last, interaction networks can be leveraged to infer functionalities. This is achieved by annotating the proteins of the network with ontologies on protein functions or biological processes from knowledgebases such as Gene Ontology [152], the Reactome Pathway database [153], Metascape [154], or the SIGnaling Network Open Resource (SIGNOR), a repository of manually annotated causal relationships between human proteins, stimuli, and phenotypes [155].

## Future directions

### Remaining technical challenges and opportunities

#### Biological and biochemical considerations

The major challenges in characterizing ECM interactions are the insolubility and large size of ECM proteins and the fact that many of them (*e.g*., collagens, laminins, thrombospondins) are multimers in their native state, and form supramolecular assemblies such as fibrils, microfibrils, anchoring fibrils, and networks in the native ECM tissue scaffold. As discussed throughout this review, very few (if any) interaction methods are available to investigate the biomolecular interactions occurring within native supramolecular assemblies. The experimental methods reviewed here are most often performed, not on full‐length proteins, due to the difficulty of purifying large matrisome proteins (a core matrisome protein is, on average, 1045 amino acid long), but on protein fragments or domains. It is thus likely that these interactions differ from those that would occur with full‐length multidomain ECM proteins. Another level of complexity in mapping the ECM interactome is the proteolytic release, upon ECM remodeling, of bioactive fragments, called matricryptins or matrikines [21] that have biological activities and thus an interaction repertoire of their own, different from those of their parent proteins. Last, ECM proteins undergo PTMs that can also modulate interactions and interaction parameters (*e.g*., affinity, stability). Future efforts should thus be geared toward devising experimental approaches using full‐length, native ECM proteins, preserving their structure and posttranslational modifications. This will necessitate optimizing the expression of recombinant ECM proteins in eukaryotic cells and developing reagents and approaches to extract ECM proteins and ECM complexes from tissues while preserving interactions. In addition, the design of binding assays preserving the architecture of ECM supramolecular assemblies would be a key step in deciphering the native ECM interactome.

#### Probing protein–glycosaminoglycan interactions

While we primarily described methods to characterize ECM PPIs, interactions between proteins and GAGs are also critical to the structure and function of the ECM. Several computational methods can be used to study GAG‐protein interactions [156, 157]. The biophysical approaches described above can also be used to characterize ECM protein–GAG interactions. For example, SPR and SPRi have been used to characterize interactions of GAGs with core matrisome proteins and their fragments [105, 158, 159], and of matrisome‐associated proteins such as the secreted factor interferon γ [160]. BLI can also be used to characterize the binding of GAGs to core matrisome and matrisome‐associated proteins [161, 162]. Furthermore, GAG–protein interactions have been characterized by ITC [163], MST [164], and QCM‐D [165, 166, 167]. High‐throughput assays, such as protein and GAG arrays, reviewed elsewhere [168, 169], or glycan‐mediated pull‐down coupled to proteomics [170], can also be used to identify protein‐GAG interactions, and several GAG‐binding proteomes have been characterized by AP‐MS [171, 172, 173]. Of note to the readers, experimentally supported protein–GAG interactions are available in MatrixDB, with a four‐fold increase in the number of protein‐GAG interactions stored in the database in the last 5 years [62, 174].

#### Moving toward applying unbiased methods to profile ECM protein interactomes

MS‐based approaches to analyze interactomic experiments do not require any prior knowledge of the nature of the interactants and are more likely to lead to new findings. Novel proteomic technologies have been developed to profile the interaction networks of intracellular proteins. They include sequential window acquisition of all theoretical mass spectra (SEC‐SWATH‐MS), a method based on the fractionation of native protein complexes and quantification by LC–MS/MS of proteins in each fraction, allows the detection of hundreds of protein complexes in parallel [175, 176, 177]. Deep interactome profiling by MS (DIP‐MS), combining affinity purification with blue‐native‐polyacrylamide gel electrophoresis separation, data‐independent acquisition with MS (DIA‐MS), and deep‐learning‐based signal processing of protein complexes, can resolve complexes sharing the same bait protein in a single experiment [178]. Lastly, the computational integration of immunofluorescence and AP‐MS has allowed the mapping of hundreds of subcellular protein assemblies in different cell types [179, 180]. These approaches should be particularly useful to probe the complexes that ECM proteins engage in during their biosynthesis and trafficking through the secretion pathway.

Importantly, the design of more biologically relevant experiments will lead to more robust datasets that can be used, in turn, to develop and train more reliable models to predict ECM PPIs and docking algorithms to characterize binding interfaces.

### A need to accelerate dissemination and curation efforts

The broad dissemination of high‐quality data via public repositories is instrumental in accelerating the pace of impactful biomedical discovery. To this aim, the International Molecular Exchange (IMEx) consortium, comprised of several interaction databases, including MatrixDB (Tables 3 and 4), oversees the manual curation of interaction data reported in publications using a set of rules adopted by the consortium and provides a unified open‐access dataset of molecular interactions [49]. We thus encourage those generating interaction data to make their datasets publicly available, together with sufficient metadata to facilitate reuse and curation efforts, as is now the norm for transcriptomic and proteomic data. Researchers who wish to do so are also invited to submit their interaction datasets to the ECM interaction database MatrixDB [62].

### ECM interactomics, a step toward novel therapeutic opportunities?

Because of their critical roles in any protein functions, any disruption of PPIs has significant consequences and can lead to diseases [181]. Conversely, targeting specific PPIs in diseases can rewire signaling, restore ECM organization, and achieve therapeutic benefits. Thus, mapping not only interactions but also interaction interfaces is critical to developing PPI‐targeting drugs of high efficacy. To achieve this, we need to obtain experimentally validated 3D structures of full‐length ECM proteins and ECM protein interaction pairs and complexes. These can serve, in turn, to improve interaction prediction and docking models and drug design of PPI inhibitors. Moreover, MS‐based approaches, such as limited proteolysis‐MS (LIP‐MS), have recently emerged as powerful methods to gain insight into protein structures and map protein–protein and protein–drug interaction sites [182, 183, 184]. It would thus be interesting to determine their applicability to ECM proteins. Ultimately, targeting a specific PPI should decrease the potential side effects associated with the targeting of a protein that may exert roles via interaction with different binding partners, in different subcellular compartments or tissues, and hence lead to more efficacious therapeutic strategies.

## Concluding remarks

A broad panel of computational tools and targeted or unbiased high‐throughput experimental approaches are now available to researchers interested in identifying and characterizing ECM protein interactions. However, ECM proteins, particularly those composing the core of the ECM scaffold, present unique biochemical properties, and there remains a critical need to develop novel techniques to probe interactions *in situ*, in the context of native insoluble ECM supramolecular assemblies. Because of their fundamental structural and functional roles, characterizing ECM PPIs also holds the potential of identifying novel targets and devising matritherapies [1] for the whole spectrum of ECM‐associated clinical presentations, including syndromes caused by mutations in ECM genes, but also cancer and fibrosis.

## Conflict of interest

The Naba laboratory holds a sponsored research agreement with Boehringer‐Ingelheim for work not related to the content of this manuscript. The authors declare having no competing interests.

## Author contributions

Conceptualization: SRB, AN. Data curation: AKB, SRB, AN. Funding acquisition: SRB, AN. Investigation: AKB, II, SRB, AN. Project administration: SRB, AN. Supervision: AN. Visualization: LL, AKB, II, AN. Writing—original draft preparation: LL, AKB, II,SRB, AN. Writing—review and editing: LL, AKB, SRB, AN.
